# Mass Flow Monitoring by Distributed Fiber Optical Temperature Sensing

**DOI:** 10.3390/s19194151

**Published:** 2019-09-25

**Authors:** Alin Jderu, Marius Enachescu, Dominik Ziegler

**Affiliations:** 1 S.C. NanoPRO START MC S.R.L., Street Oltenitei, no. 388, District 4, 041337 Bucharest, Romania; alin.jderu@cssnt-upb.ro; 2 Center for Surface Science and Nanotechnology (CSSNT), University POLITEHNICA Bucharest, Splaiul Independentei no. 313, 060042 Bucharest, Romania; marius.enachescu@cssnt-upb.ro

**Keywords:** fiber optic sensors, flow diagnostics, temperature

## Abstract

We developed a novel method to monitor mass flow based on distributed fiber optical temperature sensing. Examination of the temporal and spatial temperature distribution along the entire length of a locally heated fluidic conduit reveals heat flow under forced convection. Our experimental results are in good agreement with two-dimensional finite element analysis that couples fluid dynamic and heat transfer equations. Through analysis of the temperature distribution bidirectional flow rates can be measured over three orders of magnitude. The technique is not flow intrusive, works in harsh conditions, including high-temperatures, high pressures, corrosive media, and strong electromagnetic environments. We demonstrate a first experimental implementation on a short fluidic system with a length of one meter. This range covers many applications such as low volume drug delivery, diagnostics, as well as process and automation technology. Yet, the technique can, without restrictions, be applied to long range installations. Existing fiber optics infrastructures, for instance on oil pipelines or down hole installations, would only require the addition of a heat source to enable reliable flow monitoring capability.

## 1. Introduction

Today, distributed fiber optical sensors already find applications in fault monitoring of communications networks, structural health monitoring in buildings or bridges, shape sensing, pipeline and electrical transmission line monitoring, and intrusion detection for perimeter security applications [1,2]. Here, we present their use for thermal mass flow sensing. Flow monitoring has been demonstrated by countless different methods which frequently make use of the Bernoulli’s principle, either by measuring the differential pressure within a constriction, or by measuring static and stagnation pressures to derive the dynamic pressure. However, one of the most common techniques is thermal mass flow monitoring. Its most simple implementation, a hot wire anemometer, infers mass flow rate from the heat loss to the fluid flowing past a heated metal wire [3]. The heat loss is detected via the temperature dependent electrical resistance of the metallic heating wire itself [4]. Fiber optic implementations of hot wire anemometers have been demonstrated [5,6,7]. However, the major drawback of most hot wire anemometers is their inability to detect flow direction. Detecting direction requires two or more independent temperature sensors in the vicinity of a heat source. In an optical fiber this can be achieved using multicore fibers [8], but more common are the realization of heaters and nearby thermometers in micro-electro-mechanical-systems (MEMS) [9,10,11]. Their fabrication remains costly and complex, and assembly procedures to integrate these sensors within a fluidic conduit is challenging [12]. In this work, we present the first experimental results on flow monitoring using distributed fiber optical sensing. Fiber optical temperature sensing exploits scattering of light to measure temperature changes. Unlike Fiber Bragg gratings, which only provide information from discrete locations, distributed fiber optic sensing measures the temperature distribution along the entire length of the fiber. Without requiring any electric interfacing the fiber can simply be attached or even fully inserted into a fluidic conduit. As demonstrated in this work, distributed fiber optical temperature sensing can give a full picture of the heat flow which leads to new detection schemes for mass flow sensing. 

The most suitable distributed fiber optical temperature sensing methods are Raman optical time domain reflectometry (ROTDR) [13,14,15,16,17], Brillouin optical time domain analysis (BOTDA) [18,19,20], phase optical time domain reflectometry (Φ-OTDR) [21], and optical Fourier domain reflectometry (OFDR) [22]. Compared to Raman sensors, BOTDA can inherently provide more accurate measurements and achieve longer sensing distances [23]. The BOTDA technique, however, requires time-durations in the range of minutes per measurement. The limiting factor for Raman or Brillouin based detection is the extremely low intensity of the scattered light. Therefore, with a higher scattering intensity, Rayleigh scattering is more suitable for long-range sensing. Using Φ-OTDR, it is possible to reach quantitative temperature measurements beyond 100 km [24,25]. The resolution of those time domain reflectometry techniques is limited by the minimal duration of the light pulse (~10 ns) resulting in a longitudinal resolution of about one meter. This limit can be overcome with optical frequency domain reflectometry (OFDR), where backscattered light from the fiber is combined with light from a reference arm and the intersection of these creates an interference signal. This interference signal contains information pertaining to the precise location and magnitude of reflective events along the length of the fiber under test. Once the complex reflection coefficient is obtained in the frequency domain, the reflectivity as a function of length is obtained via the Fourier transform [22]. Coherent OFDR reaches sub-millimeter spatial resolution over tens to hundreds of meters of fiber with a temperature resolution below one Kelvin [26]. Such performance is ideal to demonstrate the concept of flow monitoring on a short fluidic conduit. However, the technique is as well applicable to gas or oil pipelines, where existing fiber optics infrastructures (ROTDR, Φ-OTDR, or a BOTDA techniques) only need to be updated with a heating element in order to enable flow monitoring. 

Here, we present first experimental results on a short range sensing system using OFDR. We compare the experimental results with finite element analysis (FEA) and demonstrate that flow rates can be extracted from the recorded temperature distribution. 

## 2. Materials and Methods 

### 2.1. Experimental Setup 

**Figure 1:**
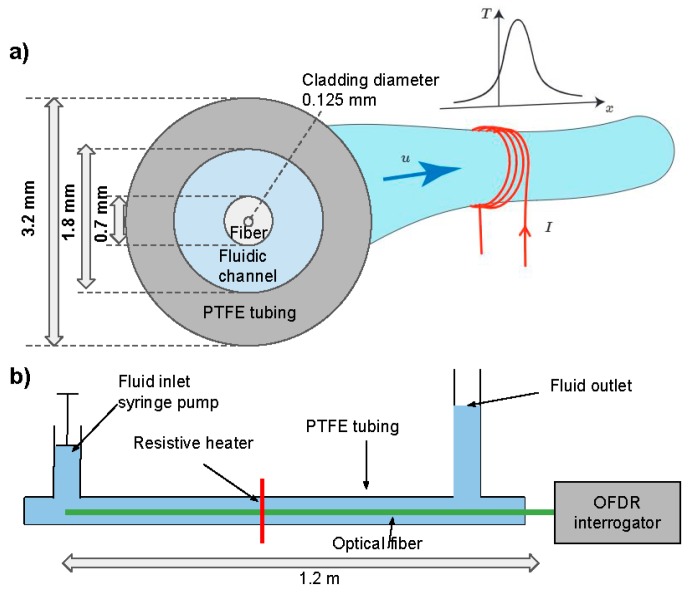
(**a**) Concept of mass flow monitoring using optical fiber temperature sensing. Joule heating in a copper wire wrapped around a fluidic conduit locally elevates the temperature of the fluid. Mass flow convectively spreads the heat downstream and the optical fiber inserted in the fluidic conduit monitors the resulting temperature distribution T(x). (**b**) Schematic illustration of the experimental setup. A syringe pump controls the fluid flow and optical Fourier domain reflectometry (OFDR) is used for distributed temperature sensing over a range of 1.2 m.

Figure 1a,b shows the concept of our short range fiber optic flow meter setup. It is realized by inserting an optical fiber into an about one-meter-long polytetrafluoroethylene (PTFE) tubing. We use a fluidic T-connector (Swivel Luer Locks) to couple the fiber into the tubing without bending or risking to apply high strain. Hot gun glue is applied to seal off the tube and prevent leakage. Before starting any temperature recording the tubing is filled with deionized water and equilibrated at ambient temperature. Joule heating of a copper wire wrapped around the tubing locally heats the wire. The temperature inside the conduit is measured using a commercially available OFDR system (ODiSI-B by Luna Technologies Inc., Roanoke, VA, USA).

### 2.2. Flow Control

To control the flow, we use a homemade syringe pump in which a bipolar stepper motor (Nema17) drives the piston of the syringe. A microcontroller controls the pulse rate and using a microstepping driver (A4988 by Pololu) a sub-micrometric linear displacement per pulse are achieved. For the inner syringe diameter of 14.5 mm we achieve a volume of 77.2 nL per pulse. Flow rates of 1, 2, 5, 10, 20, 50, 100, 200, 500, and 1000 µL/min are used in this work. As indicated in Figure 1a, the inner radius of the PTFE tube (a = 0.9 mm) and outer radius of the optical fiber (b = 0.35 mm) result in a cross-sectional area of the tube of A_tube_ = 2π(a^2^ − b^2^) = 2.16 mm^2^. Hence, the highest flow rate of 1 mL/min corresponds to an average flow velocity of 7.7 mm/s. We are asserting operation in the laminar flow regime, since for an equivalent diameter [27]:

$$D_{e}=4\frac{\pi \left(a^{2}-b^{2}\right)}{2\pi \left(a+b\right)}=2\left(a-b\right)$$

we find a Reynolds number of 9.6.

### 2.3. Heating 

To locally heat the fluid, we wrap a single loop of a 200 µm thick copper wire around the tubing and apply an electric current. We observe a linear relation between the peak temperature and the applied current, with a slope of about 10 K/A. For all experiments we use an electric current of 1.5 A resulting in a peak overheat temperature of 15 K with an ambient temperature of 27 ℃ or 300.15 K. 

### 2.4. Fiber Optical Temperature Sensing

For the temperature measurement within the 1.2 m long conduit we use coherent OFDR (ODiSI-B by Luna Technologies Inc., Roanoke, VA, USA). A baseline recording of the scatter signature is taken after equilibrating the system at ambient temperature. Then, the scatter profile is measured with heat applied and the overheat temperature is extracted from the local frequency shifts observed. All recordings of temperature are done at 8 Hz and contain 1800 data points corresponding to a lateral resolution of 0.67 mm. 

### 2.5. Finite Element Analysis (FEA)

**Figure 2:**
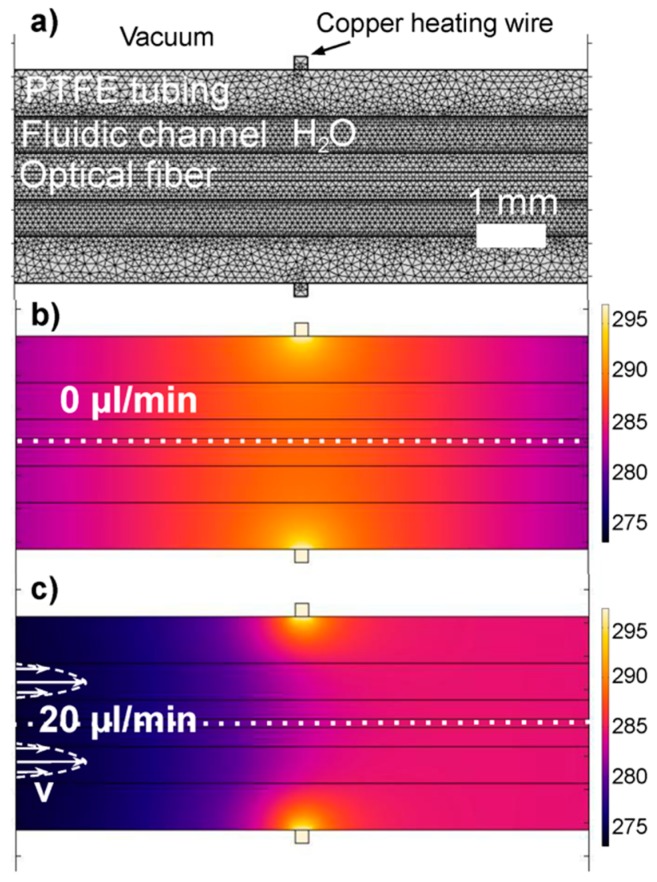
(**a**) Longitudinal cross-section of the model showing mesh size and resulting velocity profile (**b**) The resulting temperature distribution in the absence of flow is symmetrical and centered around the heater’s location. (**c**) In the presence of a flow of 20 µL/min, heat transport by forced convection shifts the temperature profile downstream. The color scale represents the absolute temperature in Kelvin.

To validate our experiments, we also perform FEA which solves the fluid dynamics coupled with heat transfer equations (Comsol Multiphysics 5.2a). The pressure and the velocity fields are solution of the Navier–Stokes equations, while the temperature is solved through the heat equation. The model assumes laminar single-phase fluid flow and uses heat transfer in fluids for the heat transfer modeling. Assuming radial symmetry of the heat in the tubing we perform a 2D simulation on a longitudinal cross-section of a 50 cm long fiber. The model’s geometry exactly matches our experimental setup, i.e., the PTFE tubing, fluidic channel and the optical fiber core (glass) with cladding (polyimide) are as shown in Figure 1a. Figure 2a shows the meshing on a close-up 9 mm long section centered on the heating element. The mesh size varies but remains smaller than 100 µm throughout the entire body. Thermal conductance (in W m^−1^ K^−1^) are as found in literature, namely 0.24 for PTFE, 0.58 for water, 1.05 for glass, and 0.12 for polyimide [28,29].

In this 2D analysis we assume a parabolic flow velocity profile in the cross-sections of the annular fluidic channel. For a heater temperature of 297 K we reach the best match with the experimentally observed overheat temperatures detected in the center of the optical fiber. Figure 2b shows the resulting heat distribution in the absence of mass flow; the heat is distributed around the heating element and shifts downstream with increasing flow velocities (see Figure 2c). A parabolic velocity profile results within the annular fluidic channel as indicated in the figure. The white dotted line indicates the center of the optical fiber where the temperature profile along the channel is recorded. 

## 3. Experimental Results 

### 3.1. Heat Diffusion: Zero Flow Experiment

**Figure 3:**
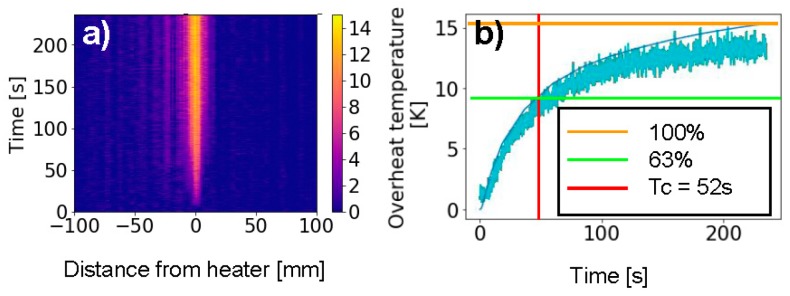
(**a**) Temporal evolution of the temperature in the vicinity of the heater for zero flow (**b**) From the evolution of the peak temperature we extract the thermal time constant of the setup.

In a first experiment we monitor the temperature buildup in absence of mass flow. As shown in Figure 2b, we would expect the heat to spread evenly on both sides of the heater location. Using OFDR as described above, we are starting simultaneously with the heating to record the temperature distribution for a duration of 235 s. As shown in Figure 3a, we observe a local heating and broadening of the temperature peak to a few millimeters over the first 100 s. The temporal evolution of the peak temperature is shown in Figure 3b and a time constant of approximately 52 s can be extracted. The position of the temperature peak precisely identifies the location of the heat source, which we define as the origin (x = 0). 

### 3.2. Forced Heat Convection: Flow Experiment

In the following we start to induce bulk fluid motion and thus force convective heat transfer. The transfer of heat away from a heater can be described using the convection-diffusion equation:

∂*T*/∂*t* = ∇ ∗ (α∇*T*) − ∇ ∗ (*VT*) + *H*(*x*,*t*) + *µ*(*x*,*t*)

where *T* is temperature, *α* is the fluid’s thermal diffusivity, *V* is the fluid’s velocity vector at any point, and *H(x,t)* is any change in temperature forced upon the system, and *µ(x,t)* indicate the losses to the environment. The response to the convection-diffusion equation is a constantly widening and shifting peak, and when convection is the dominant method of heat transfer, the peak can be tracked to derive the flow velocity. The relative dominance of convection over diffusion is described by the Peclet number *Pe = Lv α*^−1^ where *L* is length, *v* is the scalar flow velocity, and *α* the thermal diffusivity of water (*α* = 143 × 10^−9^ m^2^/s). For a Peclet number greater than one the system is dominated by convection [30,31]. For low flow rates and short distances, diffusion is the dominant method of heat transfer, and the heat will tend to dissipate before it moves over a significant distance.

**Figure 4:**
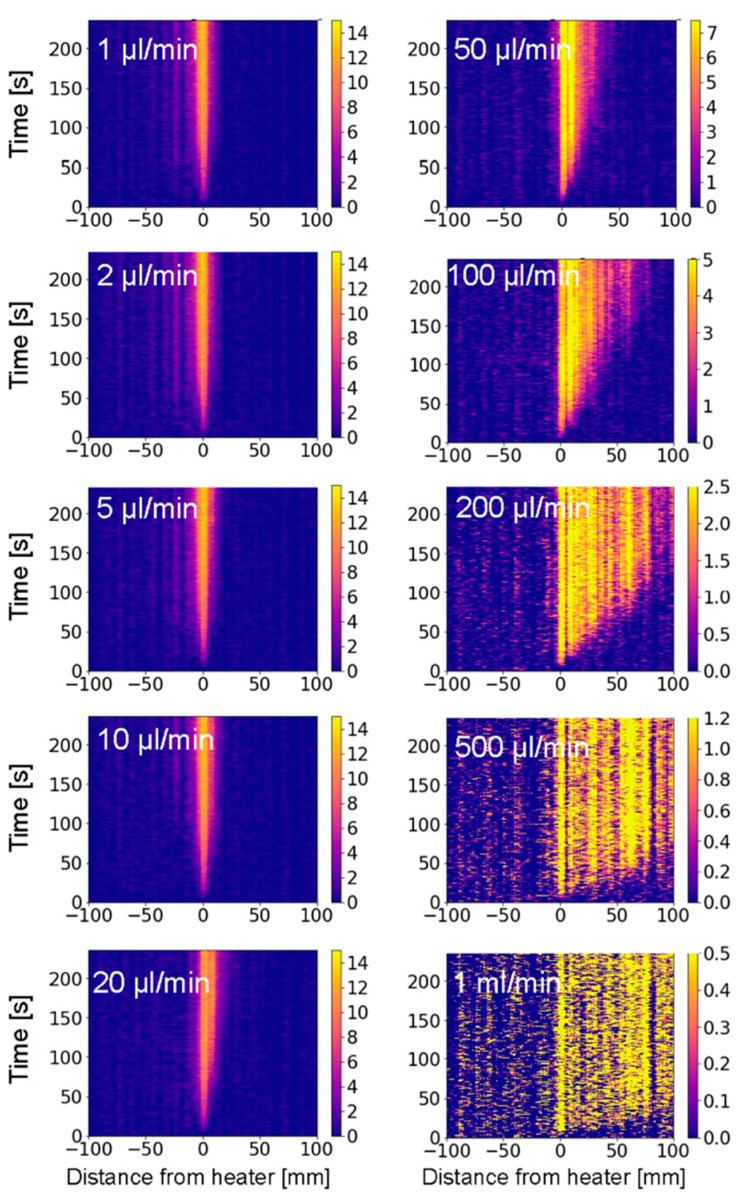
Experimentally obtained evolution of the heat distribution for the first 235 s after heating started, for flow rates ranging from 1 µL/min to 1 mL/min as indicated. Note the decrease of the temperature scale from 15 K for a flow of 1 µL/min to 0.5 K for 1 mL/min. The color scale represents the overheat temperature in Kelvin.

Figure 4 shows the temperature profiles for flow rates ranging from 1 µL/min to 1 mL/min. The experimental results for the ten different flow rates are shown on a zoomed-in 200 mm long section of the fiber. With increasing flow rates, we observe a decrease of the peak temperatures and a spreading of the heat downstream. Note the difference in color scales from 15 K for the lowest flow rate to 0.5 K for the highest flow rate.

### 3.3. Finite Element Analysis

**Figure 5:**
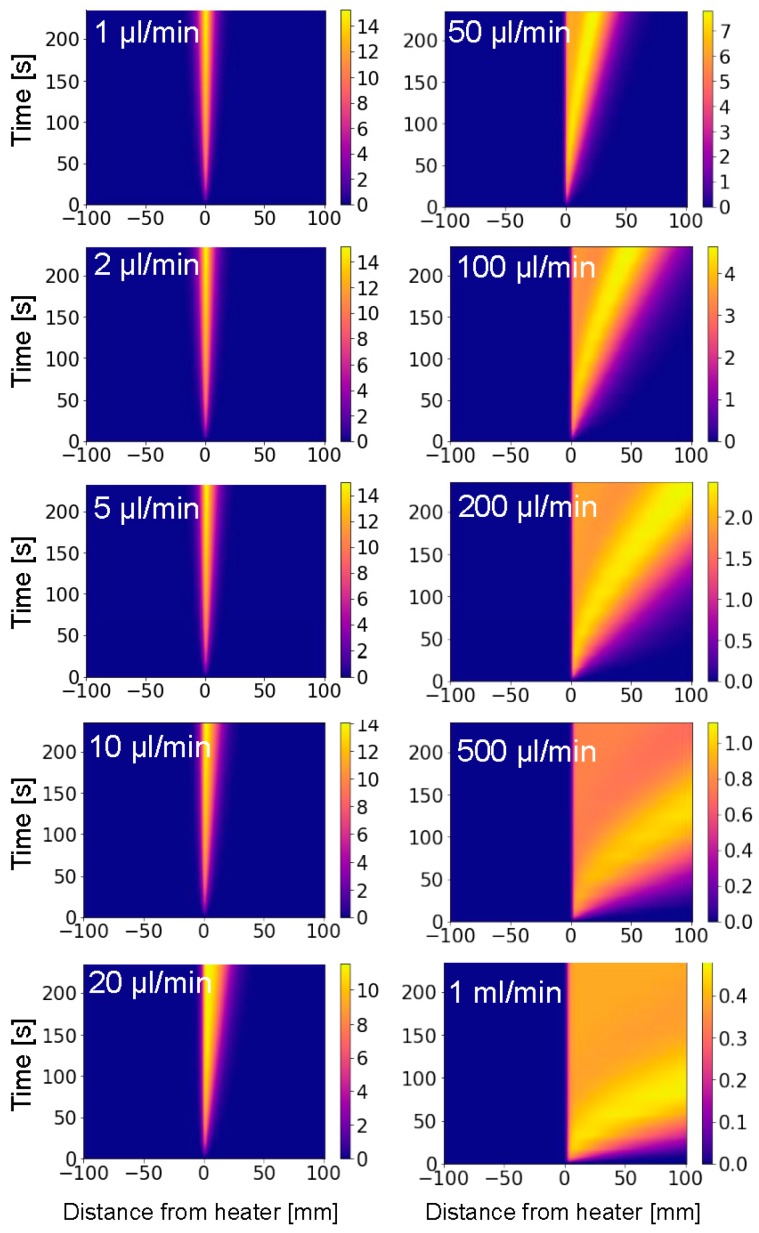
Finite element analysis (FEA) of the heat distribution for the first 235 s after heating started for flow rates ranging from 1 µL/min to 1 mL/min as indicated. The color scale representing the overheat temperature in K, is the same as for the experimental data (see Figure 4).

Figure 5 shows the data for temperature distribution under forced convection obtained with FEA for the same flow rates and geometries and materials as used in the experiment. To facilitate comparison, we use the same time scale, length scale, and color scales as in Figure 4.

### 3.4. Comparison Experiment and Simulation

**Figure 6:**
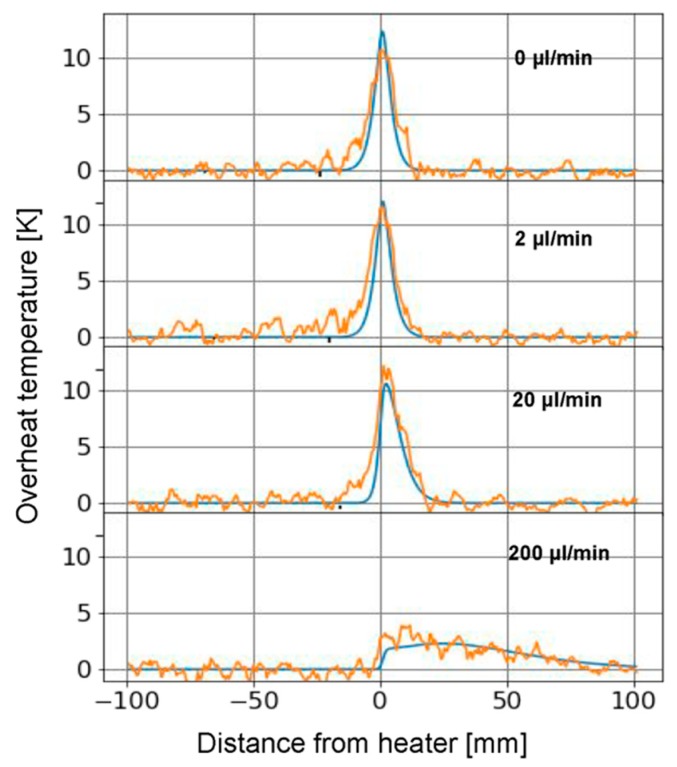
Overheat temperature vs distance from heater for raw experimental data (orange) and simulations (blue) for flow rates of 0, 2, 20, 200 µL/min as indicated. Data for t = 100 s is shown.

By comparing the two results, we observe a very good match between simulation and experimental results. Their similarity becomes more evident when plotting the overheat temperature against the distance from the heater for four selected flow rates as shown in Figure 6. The orange curves represent the raw experimental results and the blue curves the FEA data. We observe that the peak to peak noise in the experimental data is about 3 K. Some of the variations can stem from undesired motion of the optical fiber. Since the fiber was simply slid into the tubing, its position relative to the center of the tube might vary. This can lead to residual strain as well an off center placement of the sensor that can result in such temperature variations. 

### 3.5. Analysis of the Temperature Distribution

**Figure 7:**
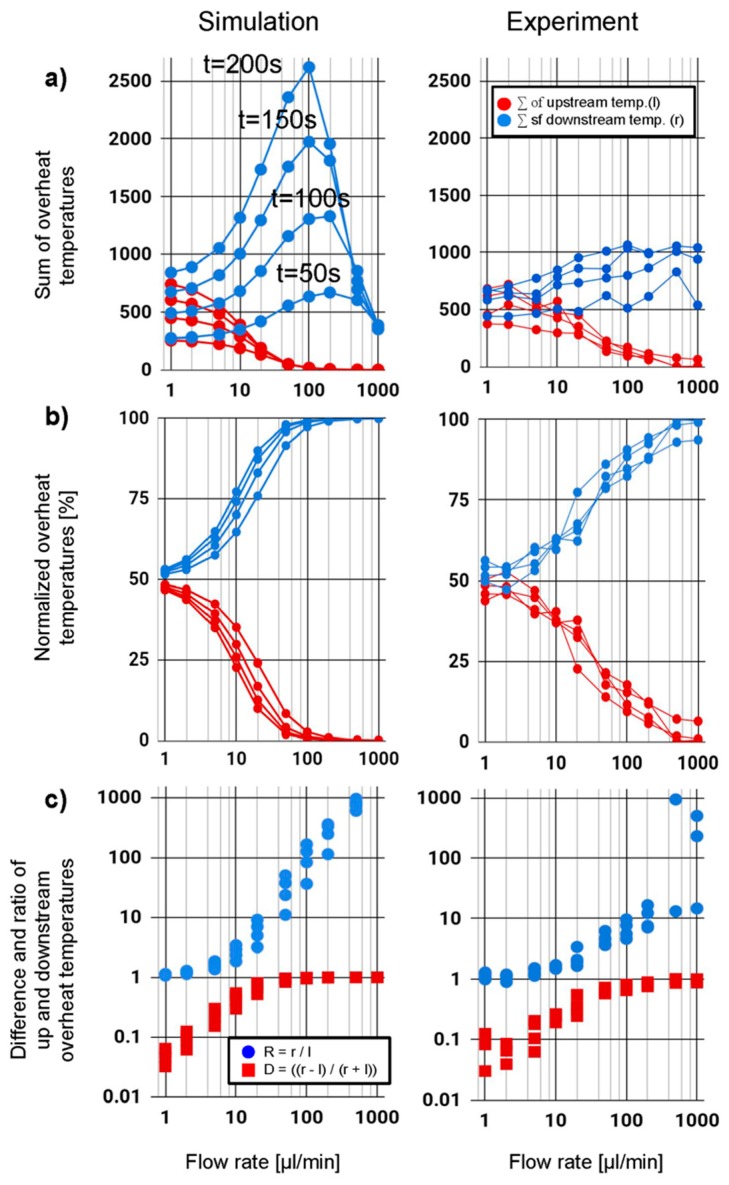
Comparison of FEA simulation (left) and experimental results (right). (**a**) Overheat temperatures for upstream (red) and downstream (blue) plotted against flow rate. The data for time t = 50, 100, 150, and 200 s is shown. (**b**) Normalized overheat temperatures (**c**) Differential *D* (red) and ratio *R* (blue) of up and downstream values. While for low flow rates (<10 µL/min) the difference is a more favorable metric, the ratio (*R* in blue) shows a better response for high flow rates (>10 µL/min).

In the following we quantify the asymmetry in the temperature distribution with respect to the heater location. To this end, we separately sum up the overheat temperatures of all data points upstream (*l*) and downstream (r) from the heater location (*x* = 0 mm):

$$l=\overset{0}{\underset{x=-\infty }{\sum }}T\left(x\right),\;r=\overset{\infty }{\underset{x>0}{\sum }}T\left(x\right),\;S=r+l$$

where *T*(*x*) are the measured overheat temperatures and *S*, the sum of all recorded overheat temperatures. Figure 7 plots the upstream temperatures (*l*) in red and downstream temperatures (*r*) in blue against three orders of magnitude in flow rates. Both results for the simulation (left) and for experimental results (right) show data taken at times t = 50, 100, 150, and 200 s. In the simulation, where no heat is leaving the system through radiative losses, heat accumulates over time until it eventually escapes as convective flow out of the section under observation (see flow rates of 200 µL/min and higher). 

In the simulation data, we observe a substantially higher accumulation of heat downstream than in the experimental data (blue curves). This difference can be explained by losses to the surrounding environment, which were simulated, but effectively lower the experimentally observed overheat temperature. 

To compensate for heat losses, we normalize both datasets by dividing *l* and *r* by their sum *S*, which results (as shown in Figure 7b). As expected for zero flow, the heat spreads evenly in both directions. Hence, the percentage of heat transferred downstream is 50%. For high flows of 1000 µL/min, however, close to 100% of the heat is transferred downstream. This measure is much less independent on the time when the data points were collected. 

In the following we introduce a normalized temperature difference (*D*) between up and downstream given by *D = (r − l)/s* (see red squares in Figure 7c). *D* is independent of the overheat temperature and increases monotonously with flow rate. For flow rates above 10 µL/min the difference signal asymptotically approaches 1. This is because in a convection dominated regime no more heat is diffusing upstream and *l* approaches zero. *D* reaches one for the non-flow condition where only diffusion contributes to heat flow and *r = l.*

A second metric is the ratio (*R*) of *l* and *r, R = (r/l) −* 1. (see blue dots in Figure 7c). *R* is one for zero flow rates and increase monotonously with flow rates. While for low flow rates (<10 µL/min) in the diffusion-dominated regime, the difference (*D*) is a more favorable metric, the ratio (*R*) shows a better response for high flow rates (>10 µL/min) where convection dominates. With the exception of few data points for very small flow of 1µL/min and very high flows both curves show a monotonous increase with flow rates, and can therefore be used for sensing. Based on our preliminary results the lower detection limits are below or 2 µL/min (32 nl/s). A detailed analysis of the system’s upper and lower detection limits is part of planned future work. These detection limits are related to the lateral and thermal resolution of the fiber optical temperature sensing method. The low detection limit combined with a large dynamic range outperforms many other flow sensing techniques. 

## 4. Discussion 

We have performed our experiments with continuous heating and demonstrated a simple method to analyze the temperature distribution to infer flow rates. Using the same setup, a more commonly used pulsed heating approach could be applicable without any restriction. Therein, heat is injected for a known short period of time and the temperature distribution is observed, i.e., the temperature distribution is measured at a known time interval after the heating pulse was applied. By taking the full temperature distribution into consideration and normalizing our method becomes independent of the heating power, geometry, and thermal conductivity of the tubing or liquid itself. A key advantage over most other fluidic sensors is that this implementation is resilient to many harsh conditions including high temperature, high pressure, corrosive media, and strong electromagnetic environments. Moreover, there are no movable parts which guarantees long-term reliability. Another advantage over other flow sensing mechanisms is that the fluid channel does not need to be interrupted to install a sensor. An optical fiber with one or even multiple heating elements can simply be retrofit to existing fluidic conduits. Each heater location turns into a flow measuring point. Optical fiber installations on pipelines can become flow monitors by the simple addition of a heating element. On the short range a direct medical application could be very low dose drug delivery. In addition to flow metering, the distributed temperature sensing also allows to monitor exo- or endothermic reactions that can occur when mixing reacting fluids. Monitoring the temperature along fluidic conduits can also be useful for quality control of process streams in the food industry, chemical industries, or to study metabolic rates in biological systems.

## 5. Conclusions

We demonstrated a novel technique to monitor the heat distribution in a fluidic conduit using internal distributed fiber optical temperature sensing. We found good agreement between experimental results and two-dimensional finite element analysis of the temperature distribution. The capability to detect bidirectional flow rates over three orders of magnitude exceeds typical ranges of commercially available flow monitoring tools. The method works for highly viscous, highly corrosive, or opaque liquids, where use of optical methods is occluded. We demonstrated this novel technique for short range sensing, however, without difficulties the method is applicable using other fiber sensing techniques, e.g., BOTDA, ROTDR, or Φ-OTDR whose range can exceed 100 km. Therefore, this technique can find use in gas or oil pipelines or any type of supply lines for chemical, medical, food or pharmaceutical industries. By the simple addition of a heating element any existing pipeline equipped with temperature or stress sensors can be upgraded to a flow monitor. Thanks to its long range, 2D or even 3D applications (where the fiber is arranged in three dimensions in space) can also be envisaged. Such advances could be used to monitor river flow rates or even water flows in the sea.
